# Passive Detection of Biological Aerosols in the Atmosphere with a Fourier Transform Instrument (FTIR)—the Results of the Measurements in the Laboratory and in the Field

**DOI:** 10.1007/s11084-012-9288-z

**Published:** 2012-06-17

**Authors:** M. I. Błęcka, M. Rataj, G. Szymański

**Affiliations:** 1 Space Research Centre of Polish Academy of Sciences, Bartycka18a, 00-716 Warsaw, Poland; 2 Faculty of Mechatronics, Warsaw University of Technology, Św. Andrzeja Boboli 8, 02-525 Warsaw, Poland

**Keywords:** Bio-aerosols, Spectroscopy, Remote sensing of the atmosphere, Astrobiology

## Abstract

Fourier Transform Infrared Radiation (FTIR) spectroscopy is one of the most powerful methods for the detection of gaseous constituents, aerosols, and dust in planetary atmospheres. Infrared spectroscopy plays an important role in searching for biomarkers, organics and biological substances in the Universe. The possibility of detection and identifications with FTIR spectrometer of bio-aerosol spores (*Bacillus atrophaeus var. globigii*=BG) in the atmosphere is discussed in this paper. We describe the results of initial spectral measurements performed in the laboratory and in the field. The purpose of these experiments was to detect and to identify bio-aerosol spores in two conditions: 1) In a closed chamber where the thermal contrast between the background and aerosols was large, and 2) In open air where the thermal contrast between the background and aerosols was small. The extinction spectrum of BG spores was deduced by comparing our measurements with models, and other measurements known from the literature. Our theoretical and experimental studies indicate that, during passive remote sensing measurements, it is difficult—but possible to detect and to identify bio-aerosol clouds by their spectral signatures. The simple spectral analysis described in the paper can be useful for the detection of various kinds of trace aerosols—not only in the Earth’s atmosphere, but also during planetary missions in the environments of other astronomical objects such as planets, comets etc. We expect that the interpretation of data from spectrometric sounding of Venus and Mars during the current missions Mars and Venus Express, and later during the Rosetta mission will benefit from our experimental work and numerical modelling.

## Introduction

Infrared spectrometric technique of the detection of main gaseous constituents, trace gases, various aerosols and dusts in the atmospheres of planets and environments of other objects (e.g. comets) in the Solar System is a well known research method. The spectrometers orbiting the Earth, Mars and Venus continuously give us new and interesting measurements to be interpreted. Envisat’s MIPAS, Sciamachy and GOMOS sensors are able to see holes in the ozone layer and the plumes of pollutants over industrial cities. Methane (CH_4_) (possibly of biological origin) in the atmosphere of Mars and molecular oxygen (O_2_) in the atmosphere of Venus have been detected using infrared spectroscopy.

There are over 120 molecular species discovered spectroscopicaly in the interstellar clouds. The most interesting one to astrobiologists is glycine, the simplest of life’s amino acids. About 10 to 30 % of the carbon in the interstellar medium is thought to be in the form of complex organic material PAH (polycyclic aromatic hydrocarbon) that matches the 3.4 μm infrared spectral feature attributed to CH bonds (Brownlee and Kress 2007). It is worth mentioning that PAHs are also present in the Martian meteorite ALH84001 (McKay et al. 1996) where microscopic forms that could be fossils of microbial life also exist.

Spectroscopy emerges as the most powerful tool available for the characterization of the composition and structure of atmospheres of exoplanets. Detection of molecules in the atmosphere of exoplanet has been demonstrated with the Hubble and Spitzer Space Telescopes for a transiting hot-Jupiter exoplanet. Water (H_2_O), methane (CH_4_), carbon dioxide (CO_2_), and carbon monoxide (CO) have been detected via infrared spectroscopy in emission spectrum of HD 189733b (Swain et al. 2008; Grillmair et al. 2007, 2008; Harrington et al. 2006). H_2_O, CH_4_, and CO_2_ may have potential biological significance, and thus their detection in a hot-Jupiter atmosphere is an important step in search for biomarkers and maybe for a simplest forms of life (Swain et al. 2009)

For most of Earth’s history, life was microscopic, and even now microorganisms dominate our planet in diverse and extreme environments (Shapiro 2007). For these reasons it is thought that if life exists in another place of the Universe, it might still be in the stage of microbial life.

FTIR spectroscopy has also been successfully applied in the laboratories for the detection, discrimination, identification, and classification of bacteria such as *Listeria*, *Escherichia coli*, *Salmonella*, *Staphylococcus*, and many others (Helm et al. 1991). FTIR spectroscopy is not only used as a method for bacterial identification, but it also provides information about bacterial metabolism, its growth phase, and it allows distinguishing between different serotypes (Davis and Mauer 2010).

An important conclusion of these briefly described results is that among various instruments selected to search for life spectrometers are well placed. No wonder therefore that in the recent years there has been significant interest in using passive infrared spectrometers to possibly detect biological substances in various environments.

We have decided to begin these new and promising studies in Poland as well. Our long-term experience (Błęcka et al. 2009, 2010) in the construction and use of FTIR spectrometers and in the interpretation of the spectrometric data from planetary missions (Mars-Express, Venus-Express, Herschel) allows us to be convinced that we can achieve interesting results for the benefit of the astrobiological community.

In this paper we concentrate on the passive detection of biological aerosols in the Earth’s atmosphere using our newly constructed FTIR spectrometer (this instrument will be described in detail in another paper). The results of our first set of measurements and a preliminary interpretation of the spectra are briefly outlined here.

In this first chapter we provide information about the preparation of endospores of *Bacillus atrophaeus* (BG) in the laboratory. In the next chapter we present information about our newly constructed FTIR spectrometer. The third chapter provides analysis of measurements performed in the laboratory testing cube. The results described in the fourth part of the paper are based on our initial spectral measurements performed in the field between 14th and 19th of April 2011. The final, fifth, chapter of this paper presents our initial conclusions, and describes our plans for the future.

## Laboratory Work on BG Spores

Endospores of *Bacillus atrophaeus var. globigii* ATCC 9372 (BG), a well-known simulant of *Bacillus anthracis* endospores, were used in this study. The endospores were obtained from seven days-old BG and purified of cell debris and the medium. Purity of the endospores was >98 %, verified by microscopic examination. Finally, BG endospores were suspended in ethanol, or in purified sterile water, and used for bio-aerosol generation. The densities of the endospore suspensions used for bio-aerosol generation inside the aerosol chamber were in the range of 10^6^–10^9^ colony forming units (CFU) per ml.

The transmittance of BG spores described above was measured at Military University of Technology and was used in the interpretation of the measured infrared spectra.

## The FTIR Spectrometer Constructed for Remote Detection of Bio-aerosols in the Laboratory and in the Field

The newly built FTIR spectrometer is a prototype of a portable field instrument intended to monitor the atmosphere. The instrument works in two spectral channels, namely: 3–5 μm and 8–12 μm atmospheric windows, with spectral resolution of about 1 cm^−1^. The spectral resolution and other measurement parameters were chosen to take into account dynamic behaviours of biological agents. Adequate high speed of both, optical path changes and data collection, is necessary. The system enables us to measure between 4 and 8 interferograms per second. Reduction of the spectral resolution allows optimizing the speed of the measurements. The spectrometer can work as an autonomous system collecting data in its mass memory. Moreover, the measurements can be controlled by software from a portable computer.

**Fig. 1:**
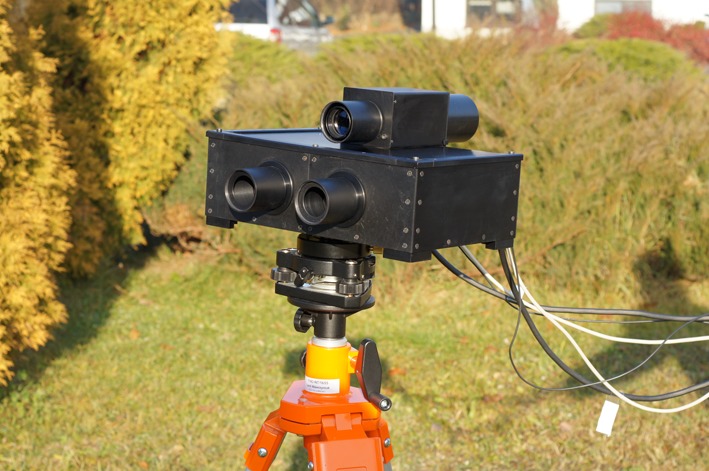
FTIR Spectrometer for the detection and identification of bio-aerosols in the atmosphere (Technical University Warsaw, Poland; Space Research Centre, Warsaw, Poland)

The spectrometer described above is shown in Fig. 1. The instrument is composed of the measurement head, a control and data acquisition unit, and a telescope allowing to select the target volume. At the front of the measurement head two entrance windows for both channels can be observed. All component parts can be mounted on the tripod. Field of view of both channels is around 1.2 deg. The diameter of the beam is 25 mm.

## Passive Remote Detection of Biological Aerosols in the Laboratory

**Fig. 2:**
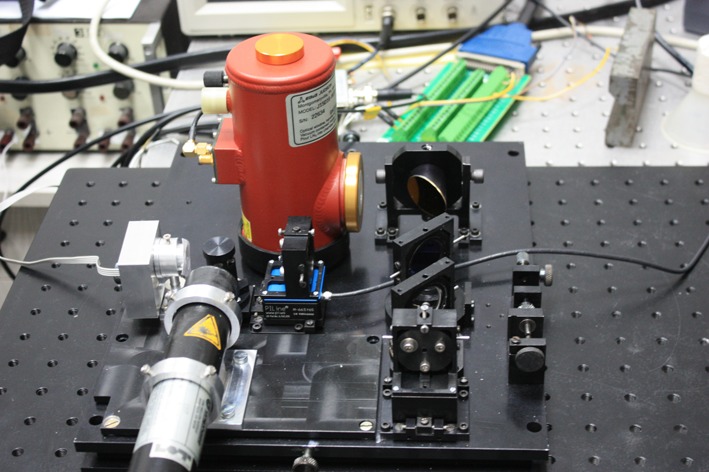
Laboratory FTIR spectrometer with nitrogen-cooled detector

**Fig. 3:**
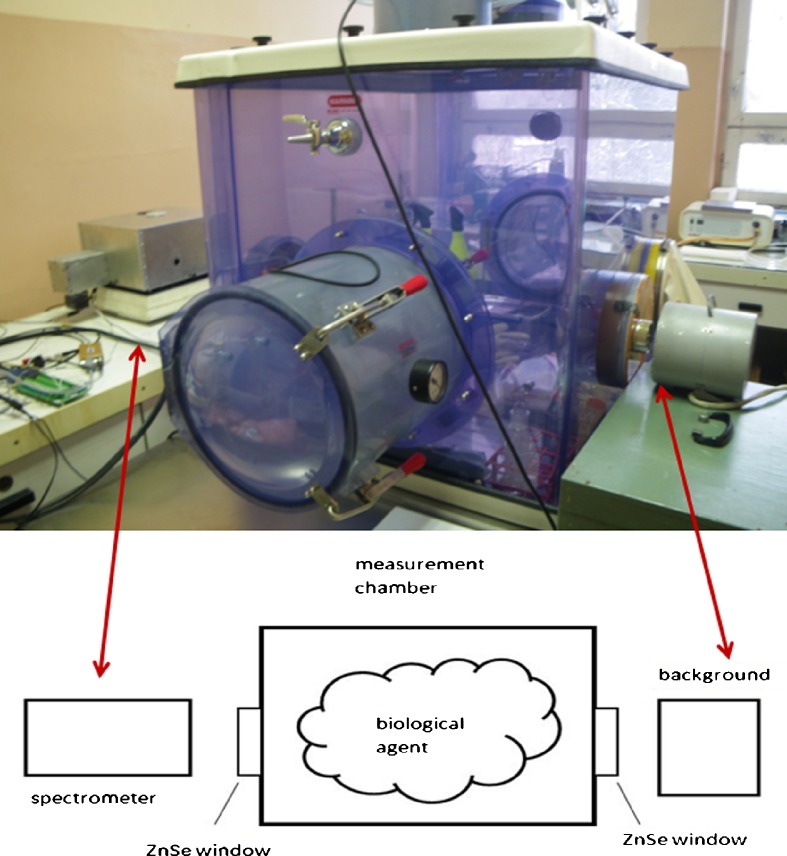
Spectrometric (FTIR) measurements of the radiance from the biological aerosols BG (scheme). The biological aerosols were injected into the sensor’s field of view. BB temperature is 85 °C

The measurements of the radiance from BG were done in the laboratory chamber using our newly constructed FTIR spectrometer. In Fig. 2 the spectrometer with nitrogen cooled detector intended for laboratory measurements is shown. The scheme of our laboratory spectrometric measurements is described in Fig. 3. Biological aerosols were injected into the laboratory cubic chamber in sensor’s field of view. The background spectra were obtained before and after the main measurements. The background in this case was a black body with various temperatures.

### The examples of radiance spectra measured in the laboratory.

**Fig. 4:**
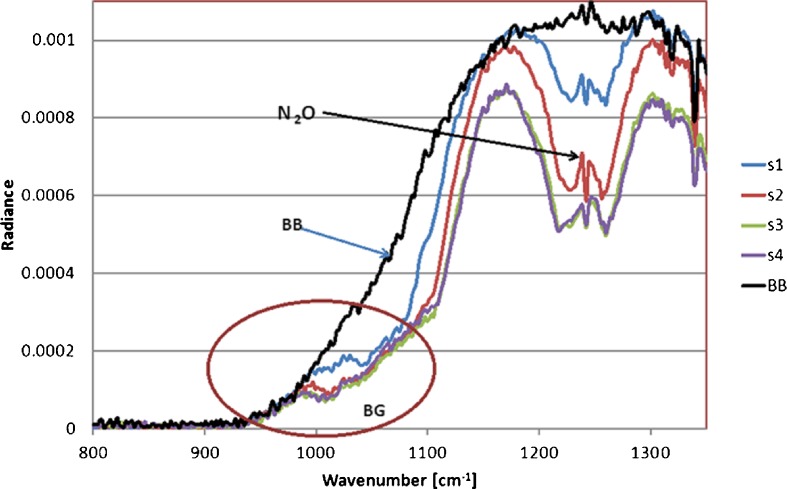
The averaged spectra measured in the cell in the laboratory. Various concentrations (s_1_–s_4_) of BG were observed (s1 ~ 3.1 × 10^4^ particles/m^3^; s2 ~ 4.1 × 10^4^particles/m^3^; s3 and s4 are >1.0 × 10^6^ particles/m^3^). The temperature of the black body is 85 °C. The y axis gives the values proportional to the radiance (arbitrary units)

In Fig. 4 the radiance spectra that were measured in the laboratory cell are shown. The results with various concentrations of BG spores can be observed. The background is a black body (BB) with a temperature T = 85 °C. The influence of BG spores is faintly visible at ~ 1000 cm^−1^. s1 to s4 means various concentration of BG; s1 ~ 3.1 × 10^4^ particles/m^3^; s2 ~ 4.1 × 10^4^particles/m^3^; s3 and s4 are >1.0 × 10^6^ particles/m^3^. The upper curve represents the radiance from the black body BB at temperature T = 87 °C. Between 1200–1300 cm^−1^ the spectral features of N_2_O present in the cell during the measurements are visible. The spectral features attributed to the biological aerosols are not well visible directly in the discussed spectra, thus their detection and particularly their identification in the atmosphere is difficult or even impossible.

For this reason we have used the simple “differential” method to prepare the spectra for a correct interpretation. Several dozen spectra were averaged. Then the differences of appropriate spectral radiances were calculated: from the cell with the bio-aerosols, and without them according to

$$ \Delta {\text{L}} = {{\text{L}}_{\text{c}}} - {{\text{L}}_{\text{t}}} $$

with

L_c_: the average radiances measured when the aerosol “cloud” was present in the cell, and
L_t_: the averaged radiances when there was no cloud in the sensor field of view

To test our methods, and to identify BG spores from the sets of spectra, we compared values ΔL with the spectral shape of the absorption coefficient of BG spores known from the literature (see Fig. 7).

**Fig. 5:**
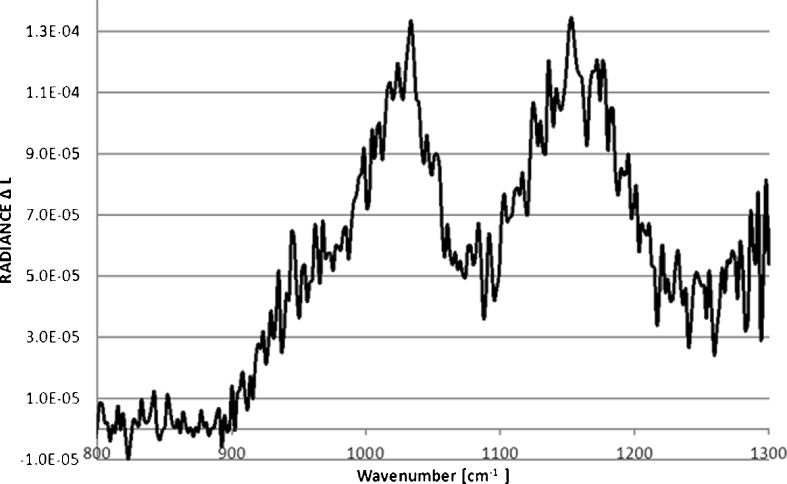
Difference ΔL of averaged radiance spectra measured in the laboratory cell

**Fig. 6:**
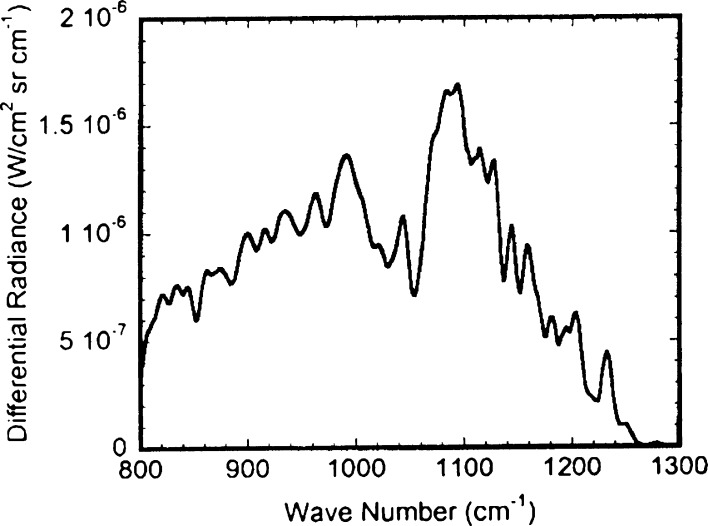
FASCODE Simulation of Differential Radiance for conditions similar to our measurements (Theriault et al. 2003)

**Fig. 7:**
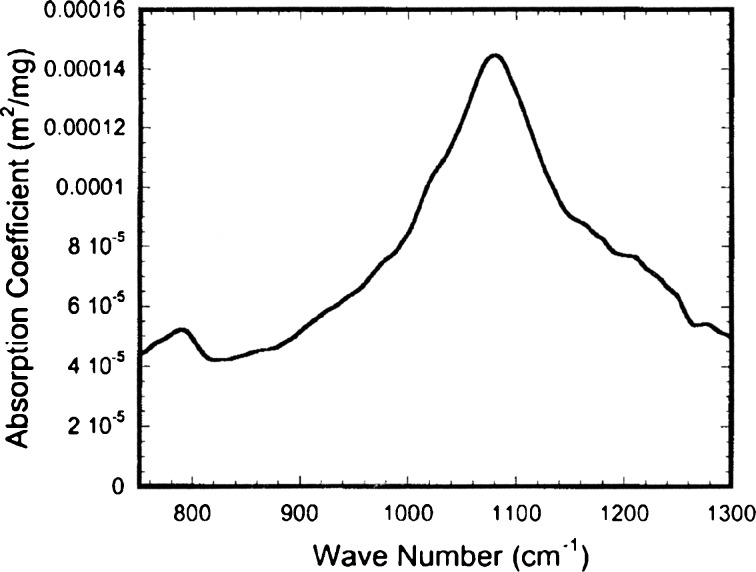
Spectral absorption coefficient of BG spores used for the detection analysis (Theriault et al. 2003)

The experimental curve ΔL shown in Fig. 5 takes the form of the extinction coefficient of BG shown in Fig. 7 with the exception of the central region where the influence of atmospheric gases is visible with variable concentrations present in the laboratory. In comparison with the results of modelling (Fig. 6) performed by FASCODE (Theriault et al. 2003) ΔL shows quite good similarity of shapes, but it is a bit shifted to larger wave numbers, probably caused by insufficiently precise calibration procedure (Fig. 7).

## Field Measurements

**Fig. 8:**
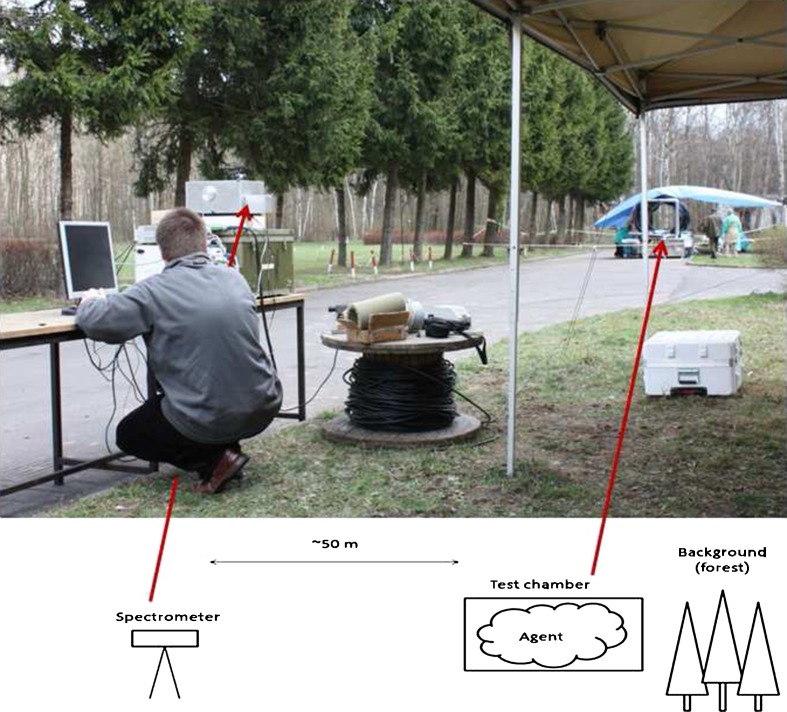
Experimental stand. Measurements were conducted along a single line of sight while the aerosol plume was disseminated into the tube in the path of the instrument

Initial passive FTIR measurements of radiance in the “outdoor chamber-tube” were performed between 14th and 19th of April 2011. Dry or aerosolized BG spores were used. The long tube was expected to isolate down-welling sky radiance. Biological aerosols were injected through the tube into sensor’s field of view. Measurements were conducted along a single line of sight while the aerosol plume was disseminated in the path of the instrument. Background spectra were obtained before and after the release. An external blackbody source was measured before and after each release to develop a preliminary calibration curve for the instrument. The experimental stand is shown in Fig. 8.

**Fig. 9:**
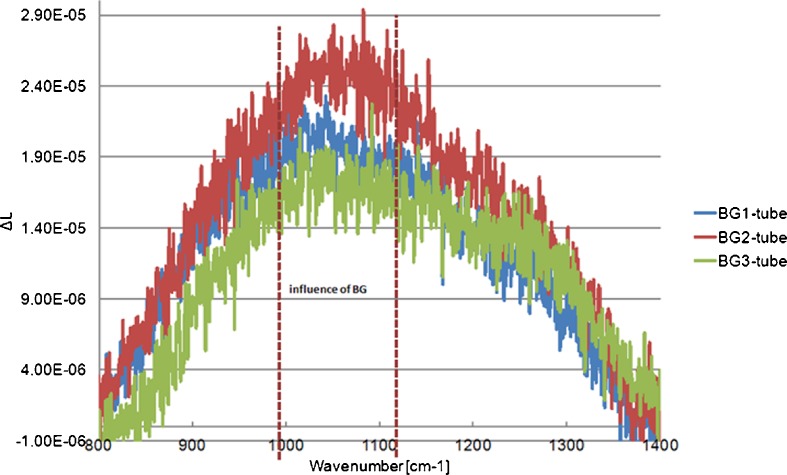
Differences ΔL of the radiances measured in the field tube. Experimental results are similar to model results in the Fig. 10. Maximal influence of BG spores appears at ~1000–1100 cm^−1^. Features from atmospheric gases (e.g. O_3_) do not appear in this case probably because of low concentrations in comparison to water vapour

**Fig. 10:**
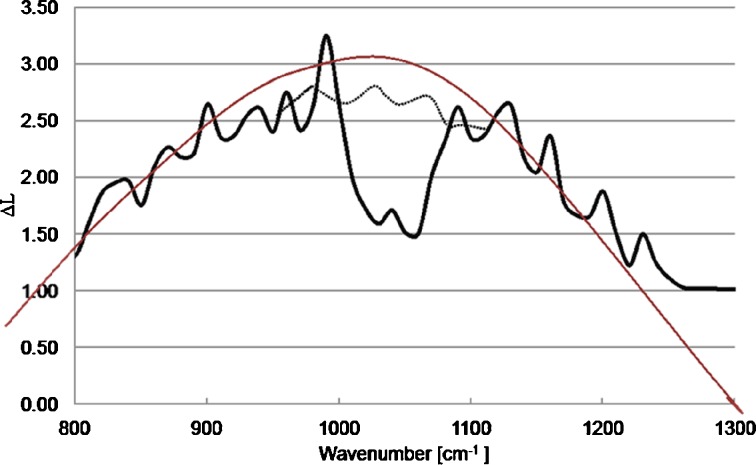
Shape of ΔL spectra from the field tube numerically simulated with MODTRAN—code (Berk et al. 1989); US Standard Model of the Atmosphere was used for calculations

Field experiments were performed in early spring (no leaves on trees, frost-covered grass) so that natural emissions of gases or smog-like aerosols were very low; also, since the path was short, tropospheric ozone was probably not present. Figure 9 shows our initial results. These experimental results are similar to model results as shown in Fig. 10. The maximal influence of BG spores appears at ~1000–1100 cm-1. Features from atmospheric gases (e.g. O_3_) do not appear in this case probably because of low concentrations in comparison to water vapour.

Figure 10 shows the ΔL spectra from the field tube that were numerically simulated with MODTRAN – code (Berk et al. 1989); US Standard Model of the Atmosphere was used for calculations. The influence of atmospheric gases is visible e.g. ozone around 1000 cm^−1^. A maximal influence of BG spores appears at ~1000–1100 cm^−1^. The smoothed shape (the brown upper curve) can be interpreted as BG absorption coefficient.

We analysed the spectra obtained in the laboratory and from the field chamber using the same methods. The spectral shapes of ΔL of the averaged spectra were similar in both cases, and the main maxima were around 1000 cm^−1^. The existing differences were probably caused by variable conditions during the measurements. Laboratory spectra are less noisy, and the influence of gases that were present in the laboratory is visible near the maximum of ΔL.

The laboratory conditions were stable during the measurements: the temperature (20 °C), pressure, and humidity around 38 %. The weather in the field was unfortunately rather bad: the temperature varied between 10 °C and 14 °C, with very high humidity. In both cases, the concentration of BG spores in the field of view of the instrument changed between successive measurements due to sedimentation by gravity and atmospheric turbulences.

## Conclusion and discussion

Preliminary results on the detection of bio-aerosols in the atmosphere performed in the laboratory and in the field are presented here. The spectral shapes of differential radiance ΔL of averaged spectra were similar in both cases, and the main maxima caused by the presence of BG spores were around 1000 cm^−1^. Our observations indicate that it is difficult, but possible to detect bio-aerosol clouds through the use of passive remote sensing by FTIR measurements. At this stage of our work, however, it is difficult to discern any type of biological substance. But we dare to believe that in the nearest future, through the use of refined spectrometric methods, we will be able not only to detect but also to distinguish between various kinds of biological particles and to identify them from their spectra (Ben-David and Ren 2003 and references therein, D’Amico 2005).

We continue our theoretical and laboratory work, and will continue it into the future. The radiometric calibration of the measurements will be repeated. But a larger collection of datasets is needed. During the next two years we will perform new tests, in the laboratory as well as in an open-air environment during various seasons, under differing weather conditions, and varying geometries of the measurements (the sensors will be positioned to view the releases at longer ranges), also with natural aerosols, kaolin dust and new biological materials. A new advanced method of spectral analysis will be also elaborated.

We consider the work presented here as the first step of our preparation for remote search of bio-substances in the atmospheres of planets during future planetary missions to Mars and Venus. The Earth’s environment is a good proving ground in this case.
